# Evaluation of Antimicrobial Properties, Cell Viability, and Metalloproteinase Activity of Bioceramic Endodontic Materials Used in Vital Pulp Therapy

**DOI:** 10.3390/jfb15030070

**Published:** 2024-03-14

**Authors:** Felipe Immich, Durvalino de Oliveira, Juliana Silva Ribeiro de Andrade, Andressa da Silva Barboza, Carlos Enrique Cuevas-Suárez, Adriana Fernandes da Silva, Wellington Luiz de Oliveira da Rosa, Álvaro Henrique Borges, Neftali Lenin Villarreal Carreno, Evandro Piva, Rafael Guerra Lund

**Affiliations:** 1 Graduate Program in Dentistry, School of Dentistry, Federal University of Pelotas (UFPEL), Pelotas 96015-560, Brazil; felipe.immich@ufpel.edu.br (F.I.); juliana.r.andrade@ufsc.br (J.S.R.d.A.); andressa.barboza@posgrad.ufsc.br (A.d.S.B.); adriana@ufpel.edu.br (A.F.d.S.); wellingtonl.fo@ufpel.edu.br (W.L.d.O.d.R.); piva@ufpel.edu.br (E.P.); 2 Graduate Program in Dentistry, School of Dentistry, University of Cuiabá (UNIC), Cuiabá 78000-000, Brazil; durvalinooliveira@gmail.com (D.d.O.); alvarohborges@gmail.com (Á.H.B.); 3 Graduate Program in Dentistry, School on Dentistry, Autonomous University of State of Hidalgo, San Agustín Tlaxiaca, Pachuca de Soto 42080, Mexico; cecuevas@uaeh.edu.mx; 4 Graduate Program in Materials Science and Engineering, Technological Development Center, Federal University of Pelotas (UFPEL), Pelotas 96010-610, Brazil; neftali@ufpel.edu.br

**Keywords:** endodontics, vital pulp therapy, dental materials, root canal therapy

## Abstract

This study aimed to evaluate the antimicrobial properties, cell viability, and matrix metalloproteinase (MMP) inhibition capacity of several endodontic materials aimed at vital pulp therapy: Pro Root MTA^®^, EndoSequence^®^, Biodentine^®^, MTA Angelus^®^, TheraCal LC^®^, and BioC Repair^®^. The materials were prepared according to the manufacturer’s instructions. Antimicrobial tests were conducted using a microcosm biofilm model, cell viability was assessed using murine fibroblasts (L929), and MMP activity was analyzed through electrophoresis. The results showed that BioC Repair^®^, Biodentine^®^, and EndoSequence^®^ exhibited similar antimicrobial properties, while MTA Angelus^®^ and ProRoot MTA^®^ had inferior results but were comparable to each other. In terms of cell viability, no significant differences were observed among the materials. EndoSequence^®^ demonstrated the highest MMP inhibition capacity. In conclusion, BioC Repair^®^, Biodentine^®^, EndoSequence^®^, and TheraCal^®^ showed better antimicrobial properties among the tested materials. The materials did not exhibit significant differences in terms of cytotoxicity. However, EndoSequence^®^ displayed superior MMP inhibition capacity.

## 1. Introduction

Vital pulp therapy (VPT) refers to a dental procedure designed to address teeth with compromised dental pulps, with the goal of preserving the pulp tissue and maintaining its health [1]. VPT involves the protection of the pulp by applying capping agents directly or indirectly on pulp tissue [2]. The advent of bioceramic materials marked a significant advancement in this emerging paradigm of endodontic therapy [3]. In the field of endodontics, bioceramics commonly employed are typically bioactive, with calcium silicate-based cements (CSCs) being the most prevalent [4]. Applied in vital pulp therapy, bioceramic materials can serve as direct pulp cappers in instances of pulp exposure due to trauma, caries, or other mechanical causes [5].

Being the first bioactive ceramic material applied in endodontics, mineral trioxide aggregate (MTA) stands as the most extensively researched bioceramic to date [6]. MTA was introduced into endodontics in 1993 by Torabinejad as a root-end filling material for endodontic surgery [7]. MTA is currently recommended in diverse clinical situations [8,9]. It is commercially available as ProRoot MTA^®^ (Dentsply Tulsa Dental, Tulsa, OK, USA). It was followed by the release of the commercially labeled MTA Angelus^®^ (Angelus Soluções Odontológicas, Londrina, Brazil). Nevertheless, potential drawbacks such as extended setting time, tooth discoloration, elevated cost, and challenging handling characteristics have emerged [10,11,12,13].

To address the limitations of MTA, various bioceramic materials have been created, with manufacturers claiming comparable characteristics to MTA but without its shortcomings [14]. Brasseler USA, (Savannah, GA, USA), introduced EndoSequence^®^ (Brasseler USA, Savannah, GA, USA), employing bioceramic technology to assert certain inconsistencies associated with conventional MTA [15]. More recently, Bio-C Repair^®^ (Angelus, Londrina, PR, Brazil) (BCR), a new silicate-based hydraulic cement, conveniently provided in a ready-for-use form, was introduced [16]. As per the manufacturer, it demonstrates outstanding consistency for easy application, serves as a barrier against microorganisms, promotes tissue healing, and does not induce discoloration [17].

Other materials have relied on polymer technology to overcome these drawbacks of MTA. Biodentine^®^ (Septodont Ltd., Saint Maur des Faussés, France) emerged as an inorganic restorative commercial cement based on tricalcium silicate (Ca_3_SiO_5_) and marketed as a ‘bioactive dentine substitute’ [18]. It was claimed as having superior physical and biological properties when compared to other tricalcium silicate cements such as MTA [18]. The enhanced handling of Biodentine is attributed to a water-soluble polymer that is incorporated to reduce the water/cement ratio, thus improving the physical properties of the cement [18,19]. Theracal LC^®^ (ThLC; Bisco Inc, Schaumburg, IL, USA) was introduced as a light activated polymerizable material, blending the favorable properties of the silicate-based component with the excellent handling characteristics of resin [20,21].

In dentistry, bioceramic materials intended for application in vital pulp therapy are anticipated to exhibit properties such as biocompatibility and bioactivity [22]. The interactions of bioceramics with surrounding tissues primarily manifest through these properties. These materials influence the proliferation, differentiation, migration, and apoptosis of various cells, including stem cells, osteoblasts/osteoclasts, dental pulp cells (DPCs)/periodontal ligament cells (PDLCs), and immune cells [5]. The outcome of wound healing and tissue repair is determined by the cellular response to bioceramics [4]. It is imperative that the material does not inflict harm upon the surrounding tissues and facilitates adequate healing. Also, evaluating cell viability can help determine whether bioceramics are safe to use in the human body [23]. Not least, bacteria are the main cause of endodontic diseases. Antimicrobial properties are an important prerequisite for the application of bioceramics in endodontics [4].

MMPs are enzymes that break down components of the extracellular matrix [24]. Gelatinases (MMP-2 and MMP-9) are associated with basement membrane degradation [25]. MMP-2 is found in normal cells, while MMP-9 is expressed in inflammatory cells [25]. Increased MMP-2 and MMP-9 levels are observed in pulpal and periapical lesions [26,27]. These enzymes contribute to dentin matrix degradation and inflammation [24]. Inhibiting MMPs could be crucial for proper healing in endodontic treatment. Assessing endodontic bioceramics’ ability to inhibit MMPs determines their effectiveness in promoting healing and preventing damage [28]. These materials are expected to have a role in tissue healing and regeneration, thereby contributing to the success of endodontic treatment [29].

Assessing the antimicrobial properties, cell viability, and metalloproteinase inhibitory potential of endodontic bioceramic materials is crucial to ensure the success of vital pulp therapy. Thus, the objective of this study is to evaluate Pro Root MTA, EndoSequence, Biodentine, MTA Angelus, TheraCal LC, and BioC Repair regarding their antimicrobial action in microcosm biofilm, cell viability in mouse fibroblasts (L929), and MMP2 and MMP9 inhibitory capacity by electrophoresis (Zymography).

## 2. Materials and Methods

The manuscript of this laboratory study has been written according to Preferred Reporting Items for Laboratory studies in Endodontology (PRILE) 2021 guidelines (Supplementary Material Figure S1). The compositions of the tested materials are described in Table 1.

### 2.1. Antimicrobial Assay

This methodology followed a previously proposed protocol [30] with adaptations. For each material tested, discs of 10 mm diameter and 1.5 mm thickness were fabricated (n = 6 per group) with the aid of a silicone matrix; all materials were manipulated following the manufacturers’ guidelines. After total curing of the specimens, they were sterilized with gamma radiation (Cobalt 60) for 2 h in a laminar flow cabinet [31].

Supragingival plaque was collected from a healthy adult volunteer, previously instructed not to perform oral hygiene for 24 h before collection, and without food consumption for 2 h. The dental plaque sample was suspended in brain heart infusion broth (BHI-Becton Dickinson, Sparks, MD, USA). Then, the discs of the tested materials were incubated in the wells of culture plates containing 1.8 mL of sterile BHI broth and 0.2 mL of inoculum, for 24 h in a microaerophilic environment (AnaeroGen; OXOID, Hampshire, UK) at 37 °C.

In the spectrophotometer (Sp22-325 at 1000 nM, Bioespectro, Curitiba, PR, Brazil), the cell density of the inoculum was adjusted, at a wavelength of 405 nM, to approximately 7.5 × 10^7^ colony-forming units per milliliter (CFU/mL) in BHI broth. Once a week, the BHI medium was changed, without replacing the microbial inoculum, until the end of the 21-day test, as biofilms with three weeks of growth or more are considered mature and may be resistant to antimicrobial agents because they have more extracellular matrix in its composition, which increases intercellular communications enabling gene exchange and the occurrence of mutations [32].

In this evaluation of the biovolume removed from the BHI medium, the disks were washed with 2 mL of saline solution, and sonicated (Sonicator UNIQUE, Indaiatuba, SP, Brazil) with a power of 30 W and amplitude of 5%, to obtain the biofilm in homogeneous suspension by 30 s.

After being sonicated, serial dilution of the bacterial suspension was performed. Then, in disposable plates containing BHI agar, the dilutions were plated, and each plate received two drops of 20 μL per dilution. Then, the plates were incubated at 37 °C for 24 h. After the incubation period, the colony-forming units (CFU/mL) were counted and the bacterial count was calculated, multiplied by the inverse of the dilution, resulting in the number of colony-forming units (CFU) per mL. The test was performed in triplicate [33]. The evaluator was blinded to the experimental groups.

The evaluation of microbiological activity was analyzed statistically by one-way analysis of variance post hoc test (ANOVA) and Tukey using SPSS software 22.9 (SPSS Inc., Chicago, IL, USA) with the significance level *p* < 0.05.

### 2.2. Cell Viability Assay/Cytotoxicity

The samples were prepared according to the manufacturer’s instructions in a laminar flow hood, previously sterilized with gamma radiation (Cobalt 60) [31]. Three disks of the tested materials (n = 3 per group) were prepared using silicone disks with 5 mm in diameter and 1 mm in thickness. These specimens were conditioned for 24 h in an oven at 37 °C, to wait for their setting, enabling the removal of the silicone molds, and subsequent removal with a sterile scalpel of the excess material.

Immediately, the specimens were stored in Dulbecco’s Modified Eagle’s Medium (DMEM, PAA, Cölbe, Germany) with 10% fetal bovine serum, penicillin, and streptomycin at 37 °C in a CO_2_ oven (5% CO_2_) for 24 h. Elution was performed using a ratio between cement surface and medium volume of approximately 150 mm^2^/mL.

For the cytotoxicity assays, the cell line of fibroblasts from L929 rats, from the Cell Culture Laboratory of the Faculty of Dentistry, Federal University of Pelotas (NCT-BIO/FOUFPel, Pelotas, Brazil), were used. The 4.5 mL graduated cryogenic tube (Techno Plastic Products, Trasadingen, Switzerland) containing the L929 cell line was brought to a temperature of 37 °C by means of partial immersion in a water bath (Biopar, Mod BM 03, Porto Alegre, Brazil) for 5 min. Then, inside a vertical laminar flow cabinet, the thawed content was added to a cell culture bottle with a surface area of 75 cm^2^ (Techno Plastic Products, Lonza, Basel, Switzerland), previously prepared with 8 mL of DMEM (Lonza, Basel, Switzerland).

The culture bottle was placed in a 5% CO_2_ oven at 37 °C and 95% humidity for a period of 3 h to promote cell adhesion to the bottom of the bottle. After this period, the culture medium containing the cryogenic protector DMSO (dimethylsulfoxide) was removed from the bottle with a sterile Pauster pipette coupled to the vacuum pump (AspiraMax Indústria de Equipamentos Médicos Ltd.a. São Paulo, Brazil). Periodically, the culture medium (DMEM) was changed from the cell line that remained stored in an incubator with a humid atmosphere, at 37 °C and 5% CO_2_, until obtaining the confluence of approximately 70% of the bottle’s cultivable surface.

Before carrying out the experiments, the number of cells in the culture bottles was determined. To determine the number (cell count), the cells were washed once in PBS and resuspended from their substrate using 4 mL of trypsin-EDTA solution (Gibco/Invitrogen, Waltham, MA, USA) for 75 cm^2^ culture bottles, for 5 min.

To neutralize the action of trypsin, 10 mL DMEM supplemented with 10% FBS and 1% antibiotic was added. Then, the total contents of the bottle were placed in a 15 mL Falcon. The cells were then centrifuged at 1200 rpm for 5 min.

After this process, the supernatant was removed, and the cells that remained at the bottom of the Falcon were diluted in 10 mL DMEM. A drop of the medium containing the cells was placed in a Neubauer chamber for cell counting in the inverted phase microscope (model AAKER). Cells with a shiny appearance and rounded shape were considered viable, and the four quadrants at the ends were not counted. The calculation was performed based on the formula where the total number of viable cells counted in the outer quadrants is multiplied by 104 (correction factor). This value is divided by the number of squares counted (in this case 4) and multiplied by the dilution factor, which is the amount of culture medium added to the cells after centrifugation. From this formula, the approximate number of cells present in each bottle was obtained. The total number of cells (TN) present in the bottle was obtained through the equation below, where DF is the dilution factor.

(1) $$TN=\frac{total\;number\;of\;viable\;cells\times DF\times 10^{4}}{4(number\;of\;squares\;counted)}$$

According to the amount of existing viable cells, the number of cells needed for cell plating was calculated to perform the cytotoxicity assays. For this test, 2 × 10^4^ cells/well are required. The cell suspension was diluted according to the number of wells required for the assay. For cytotoxicity, 96-well cell culture plates were used. Through a rule of three, the amount of cell suspension necessary for dilution in a culture medium was obtained to obtain the necessary volume in each experiment.

The cytotoxicity assay was carried out by incubating the cells with the elution of the material tested according to the international standard (ISO 10993-5, 2009) [23]. Mitochondrial activity was quantified using the MTT Assay Kit (tetrazolium salt [3-(4,5-dimethiazol-2-yl)-2,5–diphenyltetrazolium bromide (Sigma/Aldrich, St. Louis, MO, USA). A 1:1 standard ratio between cement area/medium volume of 150 mm^2^/mL was established. After a period of 24 h, for the material to set the elution was left in contact with the cells cultivated for 24 h, under 37 °C, in a humidified oven under 5% CO_2_ (Thermo Electron Corporation, Waltham, MA, USA), and then, the MTT assay for cell viability was performed. Negative control samples were treated under the same conditions; however, they were exposed to a normal culture medium.

After this exposure, the medium was aspirated and the cells washed twice with 1 mL of sterile FBS (Fetal Bovine Serum). An amount of 1 mL of MTT solution (tetrazolium salt [3-(4,5-dimethylthiazol-2-yl) 2,5 diphenyltetrazolium bromide) (Sigma/Aldrich, St. Louis, MO, USA) (0.5 mg/mL) was added to each well. After incubation for 2 h, all the MTT solution was removed, and 500 μL of sterile DMSO (Synth, São Paulo, Brazil) was added to the wells. The plates were shaken on a vortex (Biomixer, TS-2000A VDRL Shaker, Curitiba, Brazil) for 10 min and then remained immobile for another 10 min for color stabilization, protected from external light interference. After that, 100 μL of each sample was removed and transferred to a 96-well plate for subsequent reading in a microplate reader. Absorbance was determined by spectrophotometer (Thermoplate, TP-Reader, Waltham, MA, USA) and maintained at 540 nM. The test was performed in triplicate.

### 2.3. Evaluation of the Activity of Metalloproteinases

Specimens 5 mm in diameter and 1 mm in thickness (n = 3 per group) were taken from each material to be evaluated, and were suspended in a culture medium appropriate for each cell type to obtain the eluate. Each specimen used was to be incubated with at least one gel band. All evaluations were performed in vitro.

First, the separation gel was made (Table S1), placed between two 10 × 10 glass plates with the aid of a serological pipette, and then left for 30 min at room temperature to undergo complete polymerization. This gel was made with the addition of gelatin to serve as a substrate digestible by gelatinases. For the preparation of the stacking gel (Table S1), this gel was placed on top of the first one, together with the comb for the formation of the insertion wells of the samples, and stored at 4 °C overnight [34].

MMP-2 and MMP-9 were extracted from human saliva samples following stimulation. The samples underwent centrifugation at 1000 RPM for 3 min, and the supernatant was subsequently collected to isolate MMPs. The obtained samples were stored at −20 °C for future utilization. This study was approved by the Research Ethical Committee of the Pelotas Dental School (Federal University of Pelotas) on 26 October 2016, and was conducted in accordance with the principles of the 2016 Declaration of Helsinki (CAAE/UFPEL no. 57403816.2.0000.5318).

Aliquots containing enough for 11 wells of each gel were removed from the freezer (Freezer Indrel/IULT 335D, Londrina, Brazil) a few minutes before electrophoresis. Samples containing the proteins were mixed with sample buffer (4×; 100 mM Tris-HCl, pH 6.8, Vetec, Rio de Janeiro, RJ, Brazil); 4% SDS (Vetec); 20% glycerol (Vetec); and 200 μg/mL of bromophenol blue (Vetec) in a 1:5 ratio. The samples/buffer were incubated at 36 °C for 10 min and then immediately added to the wells of the stacking gel with the aid of a micropipette, with 15 μL/well being deposited.

The samples containing the enzymes were subjected to electrophoresis under non-reducing conditions (Sodium Dodecyl Sulfate, SDS), and the run was performed with the amperage fixed at 0.02 A, taking an average of 4 h (Table S2). Immediately after the end of the run, the gel was carefully removed from the glasses and transferred to a reservoir containing 2% Triton (Table S3), then left under agitation for 30 min. This procedure was performed twice to obtain enough pieces of gel for each sample of material to be tested [34].

Finally, the gel was cut into strips of approximately 1 cm, and each strip was incubated in an incubation solution (Table S4) containing EDTA (Synth, Diadema, SP, Brazil), an MMP inhibitor, NEM (Fluka Biochemika, Buchs, Switzerland), a serine protease inhibitor, and the eluates of the tested materials. The buffer containing the gel strips had its pH adjusted to 7.4, and each gel was incubated at 37 °C for 48 h to determine the gelanolytic activity of MMP [34].

As a negative control of gelanolytic activity, one strip of each polyacrylamide gel was incubated in Tris-CaCl_2_ incubation buffer for 48 h. One control was used for each assay. As a positive control, EDTA was used. Then, in one of the wells of the polyacrylamide gel, 5 μL of molecular weight standard was added, so that it could be compared with the molecular weight of the studied enzyme. The molecular weight marker BenchMark Protein LadderTM, Cat 10747-012 (Invitrogen^®^, Carlsbad, CA, USA) was used as the standard.

To identify the enzymes present in the conditioned medium, parallel inhibition experiments were performed. Two strips of each gel containing gelatin were incubated, using 45 mL Falcon tubes, in Tris-CaCl_2_ buffer at 37 °C for 48 h. In a specific tube, 0.05 mL of EDTA was added, as well as 0.05 mm of NEM (N-ethylmaleimide—Fluka Biochemika, Buchs, Switzerland).

After the incubation period, the buffer was replaced in each Falcon tube by 40 mL of 0.05% Coomassie blue (Vetec), then left for approximately 8 h (Table S5) [34].

After that, they were bleached with a methanol/acetic acid bleaching solution (Table S6) for 60 min. After the addition of the decolorizer, proteins with gelanolytic activity were visualized as negative bands. Assays were performed in triplicate [34].

The image was converted to grayscale, and the band density was qualitatively analyzed considering the presence or absence of bands. 

### 2.4. Statistical Analysis

For the cytotoxicity test, absorbance data were subjected to comparison through the ANOVA test, followed by post hoc Tukey analysis based on the sample distribution. The significance level was set at 5%. Statistical analysis for this test was carried out using the IBM SPSS software version 22.0 statistics software (SPSS Inc., New York, NY, USA).

In the antimicrobial test, data analysis was performed using the SigmaPlot12 program (Systat Inc., San Jose, CA, USA), employing one-way analysis of variance (ANOVA). The significance level for this test was established at *p* < 0.05.

## 3. Results

### 3.1. Antimicrobial Assay

**Figure 1:**
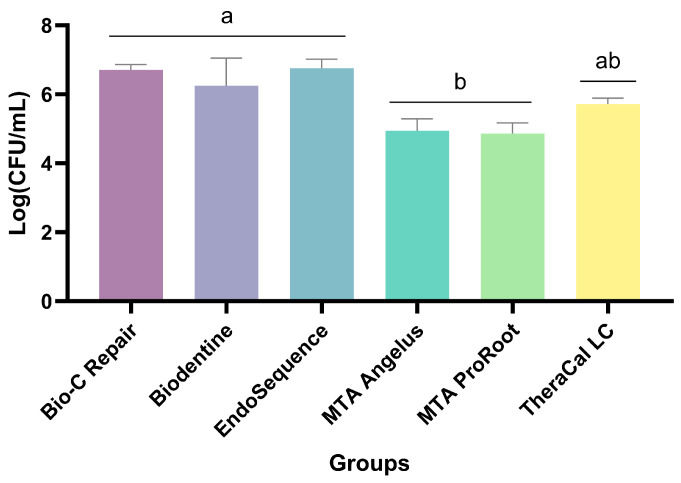
Antimicrobial action (CFU) according to each material (n = 6 per group). Mean and standard deviation. Different lowercase letters indicate significant differences (*p* ˂ 0.05).

Following the microbiological test involving a 21-day microcosm biofilm and the analysis of materials based on colony-forming units (UFCs), the results reveal similar values (*p* > 0.05) for BioC Repair, Biodentine, and EndoSequence (Figure 1). MTA Ângelus and ProRoot MTA exhibit comparable values at *p* > 0.5; however, these values are lower (*p* < 0.05) when contrasted with BioC Repair, Biodentine, and EndoSequence. TheraCal demonstrates lower values (*p* < 0.05) in comparison to BioC Repair, Biodentine, and EndoSequence, yet higher (*p* < 0.05) than the values of MTA Ângelus and ProRoot MTA.

### 3.2. Cell Viability/Cytotoxicity

**Figure 2:**
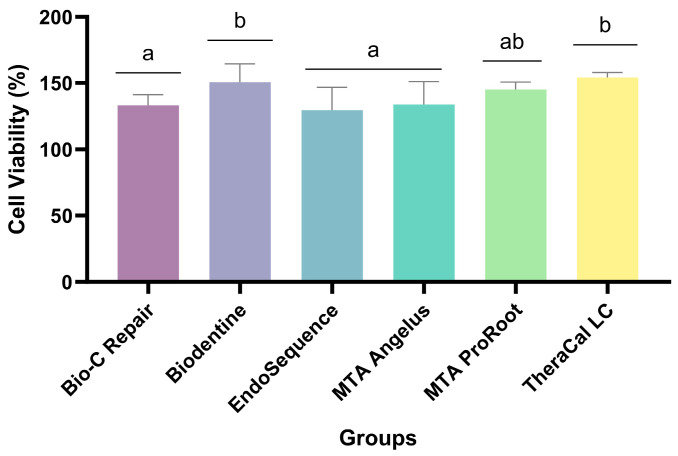
Cell viability (%) after 24 h according to each material (n = 3 per group). Mean and standard deviation. Different lowercase letters indicate significant differences (*p* ˂ 0.05).

Regarding the cytotoxicity test, (Figure 2) depicts the percentage of cell viability assessed after 24 h. The untreated group (cell control without eluate) was regarded as 100%. All groups exhibited statistical similarity to the cellular control. TheraCal demonstrated the highest cell viability at 154.27%, followed by Biodentine at 150.64%, ProRoot MTA at 145.12%, MTA Angelus at 133.84%, BioC Repair at 133.18%, and EndoSequence at 129.61%.

### 3.3. Evaluation of the Activity of Metalloproteinases

**Figure 3:**
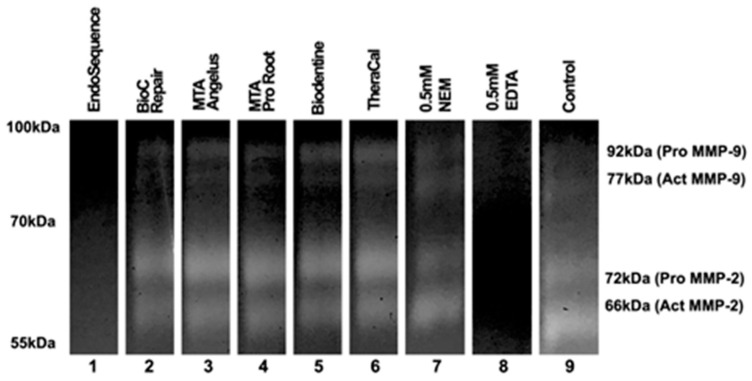
The results of electrophoresis according to each material. The results of the assay demonstrate that only EndoSequence showed an inhibitory capacity towards the gelatinolytic activity of MMP-2 and MMP-9. The original images are available in Figure S2.

Assessing the inhibition of metalloproteinases’ enzymatic activity of MMP-2 and MMP-9, as determined by the electrophoresis assay, the results indicate that only EndoSequence exhibited inhibitory capabilities (Figure 3).

## 4. Discussion

Bioceramics have been shown to exhibit remarkable bioactivity and biocompatibility, making them extensively utilized in clinical endodontic practice. Nevertheless, no bioceramic material is entirely ideal, as each has its specific limitations in practical applications [14]. There are important characteristics that materials used in endodontics need to have to promote healing. In this study, we employed the microcosm biofilm assay to evaluate the antimicrobial properties of these materials. Additionally, we assessed cell viability by conducting cytotoxicity tests using rat fibroblasts (L929), and the inhibitory effects on metalloproteinases (MMP2 and MMP9) were evaluated through electrophoresis assay (Zymography).

During the setting process, the antimicrobial and antibiofilm properties are activated by increasing the pH and the release of ions from the material [35]. The attainment of both a high-quality seal and antimicrobial properties is crucial for the success of endodontic treatment [36]. In this study, ProRoot MTA and MTA Angelus demonstrated superior antimicrobial action with lower colony-forming unit (CFU) values. Previous studies have also highlighted the antimicrobial potential of ProRoot MTA [37,38,39].

The results of our study are similar to those found in several other studies which found that MTA Angelus and ProRoot MTA exhibited greater antibacterial activity compared to Biodentine [40,41,42,43]. On the contrary, it deviates from a previous study, which indicated that Biodentine exhibited greater antimicrobial activity than ProRoot MTA [44]. Nevertheless, this assessment exclusively focused on the antimicrobial efficacy against *E. fecaelis* and did not encompass a polymicrobial culture, as in our study.

A large number of microorganisms are involved in persistent endodontic infections [45], with over 500 strains identified [46]. Certain species can have a crucial impact on a microbial community, ensuring its stability and contributing to the virulence of persistent infections [47]. Different bacterial strains vary in susceptibility to antimicrobial materials, highlighting the need for testing against multiple strains [37]. Our study focused on evaluating antimicrobial activity using biofilm microcosm, which mimics natural dental plaque in a lab setting, maintaining its complexity and biodiversity [48].

Besides antimicrobial activity, the biological compatibility of materials for vital pulp therapy is of utmost importance [49]. In the present study, all the materials achieved the minimum required recommendation of a 70% relative cell viability rate, as recommended by the ISO 10993-5 standard [50]. Moreover, all the materials showed cell viability values above 100%, indicating cell proliferation.

In terms of cell viability, TheraCal LC showed the highest cell viability, followed by Biodentine and MTA ProRoot, with no significant differences. However, other studies reported conflicting results, with TheraCal LC exhibiting higher cytotoxicity compared to MTA Angelus and Biodentine in some cases [51,52] One possible explanation for the contrasting results between these studies is that cell viability is significantly influenced by the concentration of the eluate used [53]. Different concentrations may have affected the outcomes of the studies.

Bacterial products can stimulate MMP production [25,54]. MMPs were expressed in inflamed pulps and periapical pathosis, which suggests that MMPs play an important role in pulpal and periapical inflammation and destruction of acute inflamed pulp, especially in symptomatic pulpitis [27]. Notably, increased concentrations of MMP2 and MMP9 are detected in such lesions. [26,27]. Excessive MMP production leads to tissue destruction [46]. Only EndoSequence showed the ability to inhibit MMP-2 and MMP-9 activity in the electrophoresis-based evaluation using Zymography. This inhibition is significant as it can reduce inflammation and improve pathological processes.

After dismissing the infection, promoting an anti-inflammatory environment that is conducive to tissue regeneration at the dentine-pulp interface is crucial for vital pulp therapy [55]. The key to enhancing the success of pulp vital therapy lies in the thorough assessment of pulp inflammation and the selection of suitable materials before initiating treatment [56]. In this context, bioceramic materials designed for Vital Pulp Therapy, which exhibit the inhibition of or reduction in MMPs activity, may offer advantages in terms of treatment prognosis.

While the findings indicate the suppression of MMP-2 and MMP-9 activity by EndoSequence, further investigations are required to elucidate the underlying mechanism of this inhibition. Additionally, it is essential to conduct studies specifically focused on evaluating the inhibition of MMP-9 in inflammatory cells, considering its predominant expression in these cell types.

## 5. Conclusions

This study evaluated bioceramic materials aimed at vital pulp therapy and concluded the following:

(a) All materials showed antimicrobial effectiveness, with Endosequence being the most effective, followed by Bio-C Repair, Biodentine, TheraCal LC, MTA Angelus, and MTA ProRoot.
(b) All materials promoted cell proliferation, with TheraCal showing the highest cell growth, followed by Biodentine, MTA ProRoot, MTA Angelus, BioC Repair, and EndoSequence.
(c) In the electrophoresis assay, EndoSequence successfully inhibited MMP2 and MMP9, while the other materials did not inhibit MMPs.

## Figures and Tables

**Table 1:** Composition of materials according to the manufacturer ^†^.

Material	Manufacturer	Composition
ProRoot MTA	Dentsply Tulsa Dental, Johnson City, TN, USA	Tricalcium silicate, Dicalcium silicate, Tricalcium aluminate, Bismuth oxide, Gypsum.
EndoSequence	Brasseler USA, Savannah, GA, USA	Tricalcium Silicate, Dicalcium Silicate, Zirconium Oxide, Tantalum Oxide, Calcium Phosphate Monobasic, Fillers.
Biodentine	Septodont, Saint Maur des Fossés, France	Tricalcium silicate Zirconium oxide Calcium carbonate, Calcium chloride, polymer, Calcium chloride aqueous solution and excipients.
MTA Angelus	Angelus, Londrina, Brazil	Tricalcium silicate, Tricalcium aluminate, Calcium oxide, Calcium tungstate.
TheraCal LC	BISCO, Schaumburg, IL, USA	Portland cement, Polyethylene glycol Di methacrylate, Barium zirconate.
Bio-C Repair	Angelus, Londrina, Brazil	Calcium Silicate, Calcium Aluminate, Calcium Oxide, Zirconium Oxide, Iron Oxide, Silicon Dioxide and Dispersing Agent

^†^ Composition according to manufacturers’ information (MSDS-Material Safety Data Sheet).

## Data Availability

The original contributions presented in the study are included in the article and Supplementary Material, further inquiries can be directed to the corresponding authors.

## Supplementary Materials

The following supporting information can be downloaded at: https://www.mdpi.com/article/10.3390/jfb15030070/s1. Figure S1: Flowchart according to the Preferred Reporting Items for Laboratory studies in Endodontology (PRILE) 2021 Guidelines; Figure S2: Original images of Figure 3; Table S1: Description of polyacrylamide gel reagents; Table S2: Running buffer; Table S3: 2% Triton Solution; Table S4: Incubation Buffer; Table S5: Dye Solution; Table S6: Bleaching Solution.
