# Correction: Immich et al. Evaluation of Antimicrobial Properties, Cell Viability, and Metalloproteinase Activity of Bioceramic Endodontic Materials Used in Vital Pulp Therapy. *J. Funct. Biomater.* 2024, *15*, 70

**DOI:** 10.3390/jfb15100290

**Published:** 2024-09-30

**Authors:** Felipe Immich, Durvalino de Oliveira, Juliana Silva Ribeiro de Andrade, Andressa da Silva Barboza, Carlos Enrique Cuevas-Suárez, Adriana Fernandes da Silva, Wellington Luiz de Oliveira da Rosa, Álvaro Henrique Borges, Neftali Lenin Villarreal Carreno, Evandro Piva, Rafael Guerra Lund

**Affiliations:** 1 Graduate Program in Dentistry, School of Dentistry, Federal University of Pelotas (UFPEL), Pelotas 96015-560, Brazil; felipe.immich@ufpel.edu.br (F.I.); juliana.r.andrade@ufsc.br (J.S.R.d.A.); andressa.barboza@posgrad.ufsc.br (A.d.S.B.); adriana@ufpel.edu.br (A.F.d.S.); wellingtonl.fo@ufpel.edu.br (W.L.d.O.d.R.); piva@ufpel.edu.br (E.P.); 2 Graduate Program in Dentistry, School of Dentistry, University of Cuiabá (UNIC), Cuiabá 78000-000, Brazil; durvalinooliveira@gmail.com (D.d.O.); alvarohborges@gmail.com (Á.H.B.); 3 Graduate Program in Dentistry, School on Dentistry, Autonomous University of State of Hidalgo, San Agustín Tlaxiaca, Pachuca de Soto 42080, Mexico; cecuevas@uaeh.edu.mx; 4 Graduate Program in Materials Science and Engineering, Technological Development Center, Federal University of Pelotas (UFPEL), Pelotas 96010-610, Brazil; neftali@ufpel.edu.br

**Error in Figure 3**

In the original publication [1], there was a mistake in Figure 3 of the published paper. Duplicated lanes were present in the figure. The corrected Figure 3 appears below. 

**Figure 3:**
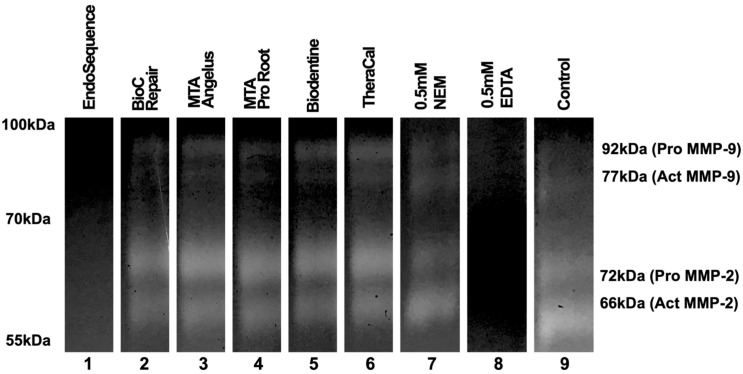
The results of electrophoresis according to each material. The results of the assay demonstrate that only EndoSequence showed an inhibitory capacity towards the gelatinolytic activity of MMP-2 and MMP-9. The original images are available in Figure S2.

**Text Correction**

There were some errors in the original publication’s Materials and Methods section, namely “2.3. Evaluation of the Activity of Metalloproteinases”. This section has been updated to clarify the experimental procedure used, as shown below:

*2.3. Evaluation of the Activity of Metalloproteinases*

Specimens 5 mm in diameter and 1 mm in thickness (n = 3 per group) were taken from each material to be evaluated, and were suspended in a culture medium appropriate for each cell type to obtain the eluate. Each specimen used was to be incubated with at least one gel band. All evaluations were performed in vitro.

First, the separation gel was made (Table S1), placed between two 10 × 10 glass plates with the aid of a serological pipette, and then left for 30 min at room temperature to undergo complete polymerization. This gel was made with the addition of gelatin to serve as a substrate digestible by gelatinases. For the preparation of the stacking gel (Table S1), this gel was placed on top of the first one, together with the comb for the formation of the insertion wells of the samples, and stored at 4 °C overnight [34].

MMP-2 and MMP-9 were extracted from human saliva samples following stimulation. The samples underwent centrifugation at 1000 RPM for 3 min, and the supernatant was subsequently collected to isolate MMPs. The obtained samples were stored at −20 °C for future utilization. This study was approved by the Research Ethical Committee of the Pelotas Dental School (Federal University of Pelotas) on 26 October 2016, and was conducted in accordance with the principles of the 2016 Declaration of Helsinki (CAAE/UFPEL no. 57403816.2.0000.5318).

Aliquots containing enough for 11 wells of each gel were removed from the freezer (Freezer Indrel/IULT 335D, Londrina, Brazil) a few minutes before electrophoresis. Samples containing the proteins were mixed with sample buffer (4×; 100 mM Tris-HCl, pH 6.8, Vetec, Rio de Janeiro, RJ, Brazil); 4% SDS (Vetec); 20% glycerol (Vetec); and 200 μg/mL of bromophenol blue (Vetec) in a 1:5 ratio. The samples/buffer were incubated at 36 °C for 10 min and then immediately added to the wells of the stacking gel with the aid of a micropipette, with 15 μL/well being deposited.

The samples containing the enzymes were subjected to electrophoresis under non-reducing conditions (Sodium Dodecyl Sulfate, SDS), and the run was performed with the amperage fixed at 0.02 A, taking an average of 4 h (Table S2). Immediately after the end of the run, the gel was carefully removed from the glasses and transferred to a reservoir containing 2% Triton (Table S3), then left under agitation for 30 min. This procedure was performed twice to obtain enough pieces of gel for each sample of material to be tested [34].

Finally, the gel was cut into strips of approximately 1 cm, and each strip was incubated in an incubation solution (Table S4) containing EDTA (Synth, Diadema, SP, Brazil), an MMP inhibitor, NEM (Fluka Biochemika, Buchs, Switzerland), a serine protease inhibitor, and the eluates of the tested materials. The buffer containing the gel strips had its pH adjusted to 7.4, and each gel was incubated at 37 °C for 48 h to determine the gelanolytic activity of MMP [34].

As a negative control of gelanolytic activity, one strip of each polyacrylamide gel was incubated in Tris-CaCl_2_ incubation buffer for 48 h. One control was used for each assay. As a positive control, EDTA was used. Then, in one of the wells of the polyacrylamide gel, 5 μL of molecular weight standard was added, so that it could be compared with the molecular weight of the studied enzyme. The molecular weight marker BenchMark Protein LadderTM, Cat 10747-012 (Invitrogen®, Carlsbad, CA, USA) was used as the standard.

To identify the enzymes present in the conditioned medium, parallel inhibition experiments were performed. Two strips of each gel containing gelatin were incubated, using 45 mL Falcon tubes, in Tris-CaCl_2_ buffer at 37 °C for 48 h. In a specific tube, 0.05 mL of EDTA was added, as well as 0.05 mm of NEM (N-ethylmaleimide—Fluka Biochemika, Buchs, Switzerland).

After the incubation period, the buffer was replaced in each Falcon tube by 40 mL of 0.05% Coomassie blue (Vetec), then left for approximately 8 h (Table S5) [34].

After that, they were bleached with a methanol/acetic acid bleaching solution (Table S6) for 60 min. After the addition of the decolorizer, proteins with gelanolytic activity were visualized as negative bands. Assays were performed in triplicate [34].

The image was converted to grayscale, and the band density was qualitatively analyzed considering the presence or absence of bands. 

The first paragraph of Section “2.4. Statistical Analysis” should be deleted. This paragraph was removed once the bands have been qualitatively analyzed for the presence or absence of bands, which indicates the noninhibition or inhibition of MMPs. This Section has been updated, as shown below:

*2.4. Statistical Analysis*

For the cytotoxicity test, absorbance data were subjected to comparison through the ANOVA test, followed by post hoc Tukey analysis based on the sample distribution. The significance level was set at 5%. Statistical analysis for this test was carried out using the IBM SPSS software version 22.0 statistics software (SPSS Inc., New York, NY, USA).

In the antimicrobial test, data analysis was performed using the SigmaPlot12 program (Systat Inc., San Jose, CA, USA), employing one-way analysis of variance (ANOVA). The significance level for this test was established at *p* < 0.05.

**Supplementary Materials**

This correction includes additional Supplementary Material: the original images of Figure 3, shown in Figure S2. 




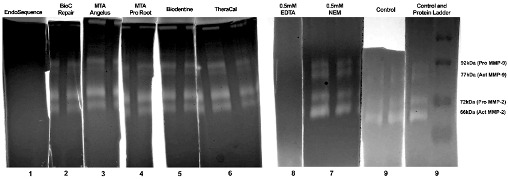


**Figure S2.** Original images of Figure 3.



As a new supplementary figure has been added, the Supplementary Materials in the paper’s back matter should be updated as shown below:

**Supplementary Materials:** The following supporting information can be downloaded at: https://www.mdpi.com/article/10.3390/jfb15030070/s1. Figure S1: Flowchart according to the Preferred Reporting Items for Laboratory studies in Endodontology (PRILE) 2021 Guidelines; Figure S2: Original images of Figure 3; Table S1: Description of polyacrylamide gel reagents; Table S2: Running buffer; Table S3: 2% Triton Solution; Table S4: Incubation Buffer; Table S5: Dye Solution; Table S6: Bleaching Solution.

The authors state that the scientific conclusions are unaffected. This correction was approved by the Academic Editor. The original publication has also been updated.

## References

[1] Immich F., de Oliveira D., Ribeiro de Andrade J.S., da Silva Barboza A., Cuevas-Suárez C.E., da Silva A.F., de Oliveira da Rosa W.L., Borges Á.H., Carreno N.L.V., Piva E. (2024). Evaluation of Antimicrobial Properties, Cell Viability, and Metalloproteinase Activity of Bioceramic Endodontic Materials Used in Vital Pulp Therapy. J. Funct. Biomater..

