# Supplementary material for: Evaluation of Antimicrobial Properties, Cell Viability, and Metalloproteinase Activity of Bioceramic Endodontic Materials Used in Vital Pulp Therapy
Source: J Funct Biomater. 2024 Mar 14;15(3):70. doi: 10.3390/jfb15030070 (PMC10971313; doi:10.3390/jfb15030070)
Supplement: Supplementary file 1 [file jfb-15-00070-s001.zip › jfb-15-00070-s001/jfb-2849873-supplementary.pdf]

Journal of
Functional
Biomaterials

MDPI

Supplementary Materials

Evaluation of Antimicrobial Properties, Cell Viability, and Metalloproteinase Activity of Bioceramic Endodontic Materials Used in Vital Pulp Therapy

Felipe Immich [1], Durvalino de Oliveira [2], Juliana Silva Ribeiro de Andrade[1], Andressa da Silva Barboza [1], Carlos Enrique Cuevas-Suárez [3], Adriana Fernandes da Silva [1], Wellington Luiz de Oliveira da Rosa [1], Álvaro Henrique Borges [2], Neftali Lenin Villarreal Carreno [4], Evandro Piva [1,4] and Rafael Guerra Lund [1,4,*]

1 Graduate Program in Dentistry, School of Dentistry, Federal University of Pelotas (UFPEL), Pelotas 96015-560, Brazil; felipe.immich@ufpel.edu.br (F.I.); juliana.r.andrade@ufsc.br (J.S.R.d.A.); andressa.barboza@posgrad.ufsc.br (A.d.S.B.); adriana@ufpel.edu.br (A.F.d.S.); wellingtonl.fo@ufpel.edu.br (W.L.d.O.d.R.); piva@ufpel.edu.br (E.P.)

2 Graduate Program in Dentistry, School of Dentistry, University of Cuiabá (UNIC), Cuiabá 78000-000, Brazil; durvalinooliveira@gmail.com (D.d.O.); alvarohborges@gmail.com (Á.H.B.)

3 Graduate Program in Dentistry, School on Dentistry, Autonomous University of State of Hidalgo, San Agustín Tlaxiaca, Pachuca de Soto 42080, Mexico; cecuevas@uaeh.edu.mx

4 Graduate Program in Materials Science and Engineering, Technological Development Center, Federal University of Pelotas (UFPEL), Pelotas 96010-610, Brazil; neftali@ufpel.edu.br

* Correspondence: rglund@ufpel.edu.br; Tel.: + 55-53-99125-7668

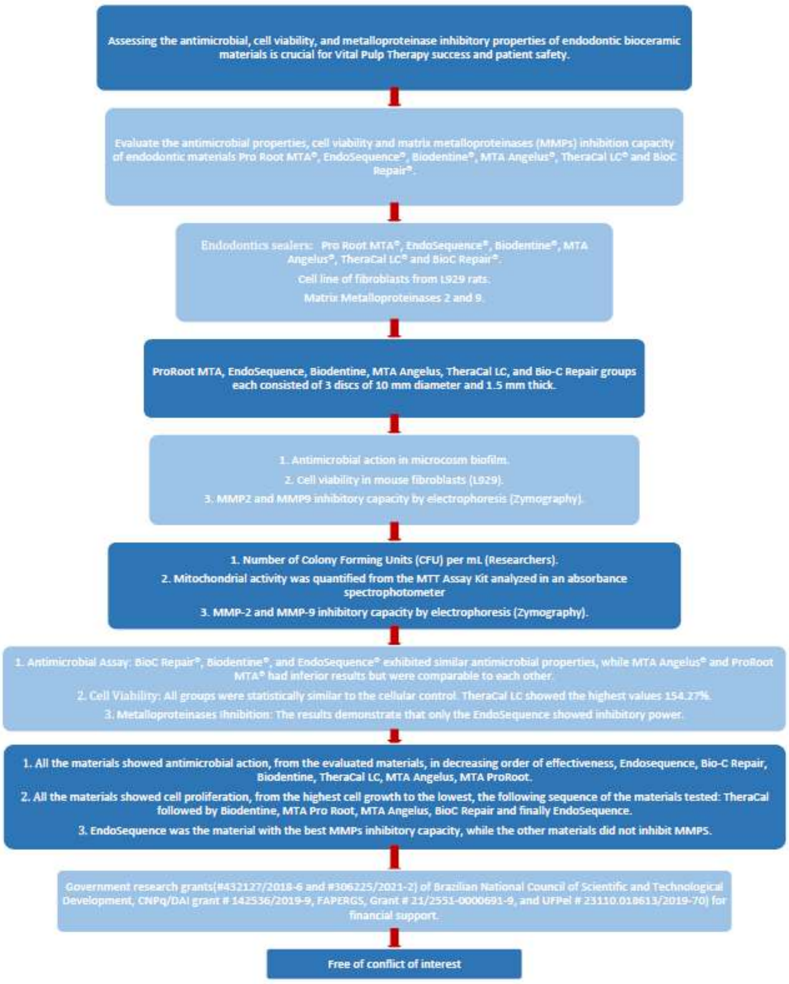

Figure S1. Flowchart according to the Preferred Reporting Items for Laboratory studies in Endodontology (PRILE) 2021 Guidelines.

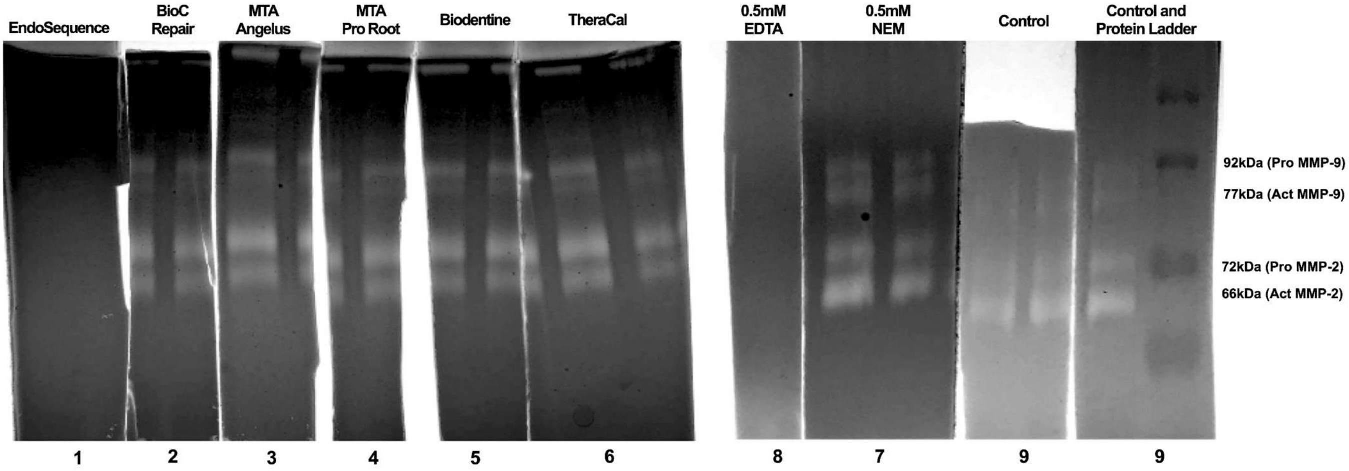

Figure S2. Original images of Figure 3.

Table S1. Description of polyacrylamide gel reagents.

Reagent/Solution	Manufacturer	Separation Gel	Starting Gel
Distilled water		3.33 mL	1.66 mL
30% Acrylamide + 0.8% Bisacrylamide	Vetec; Invitrogen	4.00 mL	0.66 mL
Separation gel buffer (Tris-HCl) pH 8.8	Vetec	2.66 mL	
Starting gel buffer (Tris-HCl) pH 6.8	Vetec		0.66 mL
Gelatin	Vetec	100.00 µL	
TEMED (N, N, N', N'-Tetramthyllenediamine)	Vetec	33.33 µL	3.33 µL
Ammonium persulfate 20%	Vetec	66.66 µL	17 µL

Vetec Indústria e Comércio, Duque de Caxias, RJ, Brazil. Invitrogen: Carlsbad, California USA.

Table S2. Running buffer.

Reagent/Solution	Manufacturer	Quantity
Glycine	Vetec	36.00 g
SDS	Vetec	2.50 g
Tris-Base	Vetec	7.55 g
Distilled water	Vetec	qsp 500 mL

Vetec Indústria e Comércio, Duque de Caxias, RJ, Brazil. † Dilute 1:4 at the time of use, no need to adjust the pH.

Table S3. 2% Triton Solution †.

Reagent/Solution	Manufacturer	Quantity
Triton-X	Vetec	10 mL
Distilled Water		500 mL

Vetec Indústria e Comércio, Duque de Caxias, RJ, Brazil. † Water must be heated.

Table S4. Incubation Buffer †.

Reagent/Solution	Manufacturer	Quantity
CaCl	Synth	1.00 mL
Tris HCl (1M)	Vetec	2.00 mL
Distilled water	Vetec	197 mL

Vetec Indústria e Comércio, Duque de Caxias, RJ, Brazil. Synth, Diadema, SP, Brazil. † Adjust pH to 7,4.

Table S5. Dye Solution.

Reagent/Solution	Manufacturer	Quantity
Methanol	Vetec	50%
Acetic Acid	Synth	25%
Coomassie blue	Vetec	0.1%
R250		qsp 500 mL

Vetec Indústria e Comércio, Duque de Caxias, RJ, Brazil. Synth, Diadema, SP, Brazil.

Table S6. Bleaching Solution.

Reagent/Solution	Manufacturer	Quantity
Methanol	Vetec	30%
Acetic Acid	Synth	10%
Distilled water		qsp 1000 mL

Vetec Indústria e Comércio, Duque de Caxias, RJ, Brazil. Synth, Diadema, SP, Brazil.
